# New WHO classification of genetic variants causing G6PD deficiency

**DOI:** 10.2471/BLT.23.291224

**Published:** 2024-06-10

**Authors:** Lucio Luzzatto, Germana Bancone, Pierre-Antoine Dugué, Weiying Jiang, Angelo Minucci, Caterina Nannelli, Daniel Pfeffer, Josef Prchal, Mahmoud Sirdah, Olugbemiro Sodeinde, Tom Vulliamy, Wanchai Wanachiwanawin, Jane Cunningham, Andrea Bosman

**Affiliations:** a Department of Hematology, University of Florence, Piazza San Marco, 4 - 50121 Firenze, Italy.; b Shoklo Malaria Research Unit, Mahidol-Oxford Tropical Medicine Research Unit, Faculty of Tropical Medicine, Mahidol University, Thailand.; c School of Clinical Sciences at Monash Health, Monash University, Melbourne, Australia.; d Department of Medical Genetics and Bioinformatics, Zhongshan School of Medicine, Sun Yat-sen University, Guangzhou, China.; e Departmental Unit of Molecular and Genomic Diagnostics, Fondazione Policlinico Universitario Agostino Gemelli IRCCS, Rome, Italy.; f Department of Experimental and Clinical Medicine, University of Florence, Firenze, Italy.; g Global and Tropical Health Division, Menzies School of Public Health, Darwin, Australia.; h Department of Medicine, University of Utah, Salt Lake City, United States of America.; i Biology Department, Al Azhar University-Gaza, Gaza, occupied Palestinian territory, including east Jerusalem.; j Department of Paediatrics, University of Ibadan, Ibadan, Nigeria.; k Centre for Genomics and Child Health, Queen Mary University of London, London, United Kingdom of Great Britain and Northern Ireland.; l Faculty of Medicine Siriraj Hospital, Mahidol University, Bangkok, Thailand.; m Global Malaria Programme, World Health Organization, Geneva, Switzerland.

Glucose-6-phosphate dehydrogenase (G6PD) deficiency is a widely distributed genetic abnormality,^1^ affecting an estimated 500 million people worldwide.^2^ Although mostly asymptomatic, G6PD deficiency can manifest clinically in three forms: (i) neonatal jaundice; (ii) acute haemolytic anaemia triggered by fava beans, infection or drugs (including some antimalarial drugs); and (iii) chronic non-spherocytic haemolytic anaemia, which is very rare. The *G6PD* gene, located on the X chromosome, is highly polymorphic and researchers have identified over 230 variants at the molecular level.^2^^,^^3^

Soon after G6PD deficiency was discovered,^4^ the World Health Organization (WHO) recognized its public health implications and summoned an ad hoc study group in December 1966. At that time, research showed that G6PD deficiency was heterogeneous,^5^ and about 20 variants had been characterized with respect to percentage residual activity (measured by the gold standard spectrophotometric assay), electrophoretic mobility, substrate affinity for G6P and NADP (nicotinamide adenine dinucleotide phosphate), activity on substrate analogues, pH dependence and thermostability.^5^ Five years later, researchers proposed a classification scheme of G6PD variants based on the above properties; this scheme, published in the *Bulletin of the World Health Organization*,^6^ became known as the WHO G6PD classification, and it was endorsed with slight changes by a WHO Working Group in 1985 (Box 1).^7^

Box 1 **WHO classification of glucose-6-phosphate dehydrogenase variants in 1985**

- Severe enzyme deficiency with chronic non-spherocytic haemolytic anaemia
- Severe enzyme deficiency (< 10% of normal)
- Moderate enzyme deficiency (10–60% of normal)
- Normal activity (60–150% of normal)
- Increased enzyme activity (more than twice normal)

WHO: World Health Organization. Source: WHO, 1989.^7^

After the human *G6PD* gene was cloned and the cDNA (complementary deoxyribonucleic acid) sequence published in 1986,^8^ it has become possible to identify the individual genetic mutations underlying previously described G6PD variants.^9^ Indeed, additional variants were discovered and classified according to the WHO G6PD classification; but in some cases, this classification was based on clinical manifestations observed in just one or two patients. Overall, the 1985 classification served its purpose well, but over the years two issues emerged as conspicuous. First, for several variants, the inter-individual variability in enzyme levels spanned across the 10% threshold that had been set to separate class II from class III variants (Box 1). Second, variants in both classes, though mostly asymptomatic, entail susceptibility to the very same clinical manifestations, namely neonatal jaundice, favism and drug-induced acute haemolytic anaemia.^2^

Thus, after half a century, a revision of the WHO classification was needed. An added stimulus came from the approval in 2018 of tafenoquine for the treatment of malaria by the United States of America Food and Drug Administration and by the Therapeutic Goods Administration of the Australian government. Because tafenoquine has a long half-life, a single dose of this medicine is as effective as a full 14-day course of primaquine in eliminating the hepatic dormant stages of *Plasmodium vivax*, thus reducing the burden inflicted by this parasite in many populations. Like primaquine, tafenoquine can produce acute haemolytic anaemia in G6PD-deficient patients, but unlike primaquine, once started it cannot be discontinued. Safe administration of tafenoquine and primaquine therefore requires testing for G6PD deficiency, whether at the point of care or otherwise.

In 2019, the WHO Genomics Initiative identified the revision of the WHO G6PD classification scheme as a priority, and the WHO Global Malaria Programme convened a technical consultation panel in January 2022 for this specific purpose. The panel considered in detail a literature review WHO commissioned,^10^ and a systematic review and meta-analysis.^11^ The panel discussed available data about the large variability of activity in hemizygous males for the most common genetic variants across the threshold of 10%, which was the divider between class II and class III variants. This variability provided a strong argument to abandon this separation. The panel recommended that class I be retained, as chronic nonspherocytic haemolytic anaemia is a rare chronic condition with well-characterized and specific clinical manifestations associated with G6PD deficiency. Class V did not need to be retained as it had been based on a single case report. The panel reached consensus that the variation in enzyme activity values for the same variant may reflect biological factors, technical factors and methodological factors, such as interference by white cells in red cell G6PD activity. The panel noted the scarcity of available data for some variants, and underscored a general need to generate more reliable data on phenotype-genotype associations for each individual variant, using standardized methods and procedures, ideally across multiple populations. Thus, for each variant, particularly the most prevalent, establishing the statistical distribution of enzyme activity values, already available for several variants, is important.^10^^,^^11^ The panel also noted that, whereas DNA analysis enables definitive identification of variants, biochemical characterization of variants including substrate affinity and stability is still important for classification, but such characterization is unfortunately often neglected and not available.

After thorough consideration, the panel recommended a revised *WHO classification of G6PD variants*, which the WHO Malaria Policy Advisory Group endorsed with minor modifications in March 2022 (Table 1). The main modification is the merger of variants formerly in class II and in class III into a single class B that comprises all the common polymorphic G6PD variants, all of which entail the risk of severe neonatal jaundice and of acute haemolytic anaemia triggered by fava beans or drugs.

**Table 1:** Current WHO classification of glucose-6-phosphate dehydrogenase variants

G6PD variant class	Median G6PD activity (% of normal)	Associated clinical manifestations
A	< 20%^a^	Chronic haemolytic anaemia
B	< 45%	Neonatal jaundice; acute haemolytic anaemia triggered by certain medicines, fava beans or infection
C	> 60%	No haemolysis
U^b^	Any	Uncertain clinical significance

G6PD: glucose-6-phosphate dehydrogenase; WHO: World Health Organization.

^a^ A variant with < 20% activity will be in class A only if it is associated with chronic haemolytic anaemia. If a variant with < 20% activity is associated with acute haemolytic anaemia induced by fava beans, drugs or infection it will be in class B; if clinical manifestations are unknown, it will be in class U.

^b^ A temporary assignment for variants for which there is currently insufficient information regarding clinical manifestations.

The WHO classification is to be applied to genetic variants of G6PD, not to individuals. This distinction is important because individuals, at a particular point in time, may have a G6PD activity higher or lower than the median value for that variant. As for the overlap in G6PD activity previously observed in men with class II and class III variants, this issue is resolved by the merger of the two classes into the new class B. The new classification does not by itself affect current WHO recommendations with respect to the use of primaquine and tafenoquine, which are based on the level of G6PD activity in each individual. Rather, the new classification, by having merged the former class II and class III, underscores that severe haemolytic anaemia, which can be life-threatening, can occur with any of the class B variants.^2^^,^^9^ In addition, the difference between class A and class B is not based on activity level, but on the coexistence of G6PD deficiency with chronic haemolysis.

Currently, no variants have been identified that in hemizygous males or homozygous females have median G6PD enzyme activity between 45% and 60%. If a variant was found in this range, it should initially be assigned to class U until compelling evidence is found that it entails susceptibility to acute haemolytic anaemia (class B) or not (class C). Some articles regarding G6PD published since 2022 have already reflected the new classification.^3^^,^^12^

Most individuals with any of the G6PD variants in class B are asymptomatic and will never know they are G6PD deficient. However, they are at risk of acute haemolytic anaemia upon exposure to oxidant agents such as antimalarial medicines primaquine and tafenoquine, for which WHO recommends G6PD testing before use. The clinical course of acute haemolytic anaemia varies in duration and severity depending on many factors: first and foremost, drug dose, but also baseline haemoglobin level and other individual variables, including the G6PD variant itself. Therefore, the clinical course of episodes of this anaemia varies, and the range of severity will not be the same with all variants that are in class B. The episodes of acute haemolytic anaemia may tend to be more common and more severe for variants with a lower mean activity (such as G6PD Coimbra and G6PD Mediterranean variants) than with a higher mean activity (such as G6PD Seattle and G6PD Kalyan-Kerala variants).

The G6PD activity characteristic of each G6PD variant has been appropriately assessed from hemizygotes and from the rare homozygotes.^10^^,^^11^ A separate issue is that of heterozygous females who, as a result of X-chromosome inactivation, have red cell mosaicism, and whose intermediate G6PD level reflects the proportion of their G6PD deficient red cell population. Since X-inactivation occurs at random very early in embryonic life, this proportion ranges from nearly 0% to nearly 100%. This variability, observed with any given G6PD variant, may override any inter-variant difference. For instance, a G6PD Seattle heterozygous female having 80% G6PD-deficient red cells may develop more severe acute haemolytic anaemia than a G6PD Coimbra heterozygote having 20% G6PD-deficient red cells, although the mean enzyme activity associated with G6PD Seattle is much higher than that associated with G6PD Coimbra.

The remarkable polymorphism of *G6PD* has contributed to understanding how individual mutations affect enzyme function and their roles in human evolution under malaria selection. WHO recommends that research on G6PD deficiency continues so that the properties of known and new variants are fully characterized.
